# Cardiovascular Risks in Autoimmune and Inflammatory Diseases

**DOI:** 10.3390/jcm15176914

**Published:** 2026-09-07

**Authors:** Elena Rezus, Ciprian Rezus

**Affiliations:** 1 Department of Rheumatology and Rehabilitation, Grigore T. Popa University of Medicine and Pharmacy, 700115 Iasi, Romania; 2 I Rheumatology Clinic, Clinical Rehabilitation Hospital, 14 Pantelimon Halipa Street, 700661 Iasi, Romania; 3 III Internal Medicine Clinic, “St. Spiridon” County Emergency Clinical Hospital, 1 Independence Boulevard, 700111 Iasi, Romania

Autoimmune and inflammatory diseases are characterized by complex pathophysiological mechanisms that promote systemic inflammation and multi-organ involvement. Persistent disease activity and inadequately controlled inflammation, whether due to insufficient treatment or treatment failure, may contribute to cardiovascular complications. Rheumatoid arthritis (RA) and systemic lupus erythematosus (SLE), among other immune-mediated inflammatory diseases, are associated with an increased risk of cardiovascular disease. A comprehensive approach to these conditions may support earlier cardiovascular risk assessment and management, with the potential to reduce long-term morbidity and mortality [1,2,3,4,5,6,7,8,9,10,11,12,13,14,15].

The new Special Issue in the Immunology & Rheumatology Section of the Journal of Clinical Medicine, entitled “Cardiovascular Risks in Autoimmune and Inflammatory Diseases”, addresses clinically relevant aspects of cardiovascular risk in immune-mediated inflammatory disorders, including risk assessment and the potential cardiovascular effects of therapy. The issue comprises eight original articles and two reviews covering psoriatic arthritis, SLE, RA, Takayasu arteritis, familial Mediterranean fever (FMF), idiopathic inflammatory myopathies (IIM), insulin resistance, and subclinical atherosclerosis.

In a cross-sectional study of 250 patients with psoriatic arthritis, Charca et al. [16] investigated markers that may enhance cardiovascular risk stratification beyond SCORE2. The assessment included carotid and femoral ultrasonography, as well as abdominal radiography. The authors evaluated hyperuricemia as a potential cardiometabolic risk enhancer and examined its association with multisite vascular plaque, aiming to improve cardiovascular risk classification in this patient population.

SLE is a chronic autoimmune disease characterized by systemic inflammation, potential multiorgan involvement, and cumulative organ damage. In a prospective study, including 88 patients with SLE, Richter et al. assessed cardiovascular comorbidities, traditional cardiovascular risk factors, and SLE-related risk factors [17]. The study also explored associations between these features and interleukin-6 (IL-6), an inflammatory marker that may contribute to a more comprehensive cardiovascular risk profile. Higher IL-6 concentrations in patients with selected comorbidities suggest a potential relationship between this cytokine and cardiovascular risk [1,2,3,4,5,6,7,8,9].

Deseatnicova et al. conducted a cross-sectional study of women with RA receiving methotrexate treatment and age-matched controls [18]. The investigators assessed carotid intima–media thickness (IMT), clinical characteristics, and immunological variables, including anti-citrullinated protein antibodies and the CD4/CD8 T-cell ratio. This integrated clinical, vascular, and immunological assessment aimed to identify factors associated with subclinical atherosclerosis and cardiovascular risk in RA.

In a seven-year prospective study involving 100 patients without a history of cardiovascular or cerebrovascular disease, Di Lisi et al. investigated the relationship between markers of preclinical atherosclerosis and the subsequent development of cardiovascular disease [19]. Participants underwent assessment with color Doppler ultrasonography of the supra-aortic trunks, IMT measurement, beta stiffness index, pulse-wave velocity, and echocardiography. Cardiovascular risk factors, metabolic syndrome, and insulin resistance were also evaluated. These data may help clarify the role of subclinical vascular markers in long-term cardiovascular risk assessment and screening strategies [11,20,21,22].

Kaymaz-Tahra et al. performed a retrospective, non-interventional observational study including 77 patients with Takayasu arteritis [23]. The authors evaluated the association between iliofemoral arterial involvement on PET/CT and demographic, clinical, and imaging characteristics, with particular attention to traditional cardiovascular risk factors. Disease activity, inflammatory markers, imaging findings, and treatment history—including immunosuppressive therapy and glucocorticoid exposure—were assessed. Such data may improve understanding of the relationship between atherosclerotic risk factors and vascular involvement in Takayasu arteritis.

FMF is an autoinflammatory disease that may be associated with systemic complications, including vascular dysfunction. In a cross-sectional study of 97 colchicine-treated patients with FMF and 81 controls, Duman et al. investigated the association between colchicine treatment and ventriculoarterial coupling (VAC) [24]. Colchicine dose was included in the analysis. VAC was assessed using echocardiographic measurements and non-invasive brachial blood pressure, allowing calculation of the arterial elastance-to-end-systolic elastance ratio. These findings may contribute to the characterization of cardiovascular alterations in FMF and support future risk-stratification research.

Popescu et al. conducted a prospective, single-center observational pilot study including 48 patients with RA treated with Janus kinase inhibitors (JAKi) [25]. Clinical, inflammatory, lipid, and vascular variables were evaluated. Vascular assessment included carotid IMT, carotid plaque detection by high-resolution ultrasound, and the ankle–brachial index. Patients were assessed at baseline and after 12 months of treatment. The study examined longitudinal changes in these parameters and explored potential cardiovascular implications of JAKi therapy in RA [26,27,28,29].

IIM comprises a heterogeneous group of autoimmune disorders characterized primarily by muscle involvement, elevated muscle enzyme levels, electromyographic abnormalities, and the presence of myositis-specific or myositis-associated autoantibodies. Szinay et al. studied 80 patients with IIM, evaluating cardiovascular risk, atherosclerosis, and disease activity [30]. The investigators used SCORE2, carotid Doppler ultrasonography, IMT measurement, and biomarkers to characterize cardiovascular risk. Following the recommendation of lipid-lowering therapy, treatment efficacy and adverse events were evaluated over six months. The authors also assessed changes in cardiovascular risk category after treatment.

RA is a prevalent chronic autoimmune disease associated with an increased risk of cardiovascular disease. In the first review included in this Special Issue, Oiegar and Pop examined the relationship between inflammation, traditional cardiovascular risk factors, cardiovascular complications, and risk assessment in RA [31]. The review discusses mechanisms involved in cardiovascular events, common cardiovascular manifestations, available risk scores, and relevant European Alliance of Associations for Rheumatology (EULAR) recommendations.

Staveri et al. provided a narrative review of cardiovascular risk stratification in SLE, highlighting the principal cardiovascular complications associated with this systemic disease [32]. The review synthesizes laboratory and imaging findings and proposes an integrated approach to cardiovascular disease prediction. The suggested framework incorporates clinical, laboratory, genetic, and imaging parameters, emphasizing the value of imaging in individualized cardiovascular risk assessment.

Overall, the contributions in this Special Issue highlight the systemic consequences of autoimmune and inflammatory diseases, particularly their cardiovascular burden. They emphasize the need for early, comprehensive, and repeated cardiovascular risk assessment, integrating traditional risk factors with disease-related inflammation, treatment exposure, biomarkers, and vascular imaging where appropriate [6,7,10,11,12,14,20,21,22]. These studies provide a valuable basis for future research aimed at improving cardiovascular prevention and treatment strategies in patients with immune-mediated inflammatory diseases.
